# Nursing students' experience of incivility behaviours and its correlation with their nursing professional values: A cross‐sectional descriptive study

**DOI:** 10.1002/nop2.1288

**Published:** 2022-07-20

**Authors:** Vahid Naseri, Mahsa Boozari Pour, Foroozan Atashzadeh‐Shoorideh, Payam Emami

**Affiliations:** ^1^ Student Research Committee, School of Nursing & Midwifery Shahid Beheshti University of Medical Sciences Tehran Iran; ^2^ Department of Medical Surgical Nursing, School of Nursing and Midwifery, Shahid Modarres Hospital Shahid Beheshti University of Medical Sciences Tehran Iran; ^3^ Department of Psychiatric Nursing and Management, School of Nursing and Midwifery, Shahid Labbafinezhad Hospital Shahid Beheshti University of Medical Sciences Tehran Iran; ^4^ Department of Emergency Medical sciences, Faculty of Paramedical sciences Kurdistan University of Medical Sciences Sanandaj Iran

**Keywords:** Incivility behaviour, nursing professional values, nursing students

## Abstract

**Aims:**

The purpose of this study was to determine the relationship between experienced incivility behaviours and professional values in the clinical setting and also the factors influencing incivility and professional values**.**

**Design:**

Cross‐sectional descriptive study.

**Methods:**

Data gathering tools used in the study includes demographic characteristics, incivility behaviour and nursing professional values. Data were analysed using IBM SPSS version 21.

**Results:**

The findings showed that incivility behaviours had a statistically significant negative correlation with professional values (*r* = −.150, *p* = .003), so that the level of incivility behaviours experienced by students was low (1.76 out of 5). This was while the level of professional values was moderate to high among students (3.72 out of 5). Due to the negative and statistically significant correlation between incivility behaviours experienced by students and nursing professional values, it is necessary that the officials of teaching hospitals inform nurses about incivility behaviours. Considering the negative and statistically significant correlation, it can be concluded that the reduction in nurses' incivility behaviours as patterns of the nursing profession causes nursing students to better acquire and internalize nursing professional values in the clinical setting. Nurse educators should also try to communicate with nursing administrators to exchange information about nurses' civil and incivility behaviours perceived by students.

## 1 INTRODUCTION

Incivility behaviours have been identified as one of the major problems in the clinical setting of the nursing profession. In clinical settings, nursing students face many challenges in communicating with other students, educators and nursing staff. These challenges shape their professional and social character (O’Mara, McDonald, Gillespie, Brown, & Miles, 2014). In addition to learning in a clinical setting, the nursing students can practice the process of joining a social organization. However, nursing students experience a lot of stress in the face of these unfamiliar settings and different situations (Wallace, Bourke, Tormoehlen, & Poe‐Greskamp, 2015). The experience of incivility behaviours in the clinical setting by nurses is one of the stressors for nursing students (Kim, 2018; Wallace et al., 2015). Nurses’ incivility behaviour is increasing among nursing students and is one of the problems affecting nursing education in different countries (Clark et al., 2010; Natarajan, Muliira, & van der Colff, 2017). The incivility behaviour, defined as disrespectful speech or action, includes abusive remarks, verbal abuse and violent behaviour that can undermine one’s self‐confidence and cause one to doubt one another or oneself (Birks, Budden, Biedermann, Park, & Chapman, 2018; Peters, 2014; Peters, McInnes, & Halcomb, 2015). In the clinical settings, the nursing students experience the incivility behaviours such as ignorance, indifference, blame, rude speech and abusive gestures from nursing staff, clinical educators or patients (Ahn & Choi, 2019). Previous studies have shown that experiences of nursing students’ incivility behaviour can have a serious impact on their perceptions of stress, depression, burnout and low self‐confidence, and even lead to early retirement (Ahn & Choi, 2019; Babenko‐Mould & Laschinger, 2014; Budden, Moulton, Harper, Brunell, & Smiley, 2016; Clarke, Kane, Rajacich, & Lafreniere, 2012; Laschinger & Fida, 2014). Such experiences not only cause stress and burnout but also have other negative effects on clinical education and prevent students from learning (Anthony, Yastik, MacDonald, & Marshall, 2014; Hakojärvi, Salminen, & Suhonen, 2014). Other studies have shown that the incivility behaviours can lead to unsafe working conditions, reduced quality of care and patient safety, reduced job satisfaction and increased medical costs (Clark, Olender, Cardoni, & Kenski, 2011; Laschinger, 2014; Rad, Ildarabadi, Moharreri, & Moonaghi, 2015). The teaching of professional behaviour, and the formation of a professional identity, is considered by many to be central to the purpose of nurse education (Guo, Shen, Ye, Chen, & Jiang, 2013). Adopting professional behaviours has a conspicuous role to development of professional identity (Del Prato, 2013). The rationale for exploring incivility in nursing is grounded in the need to ensure that student nurses enter the nursing profession equipped with the professional behaviours expected of them and that patient care will be delivered by polite, respectful and caring nurses. The risk of failing to address incivility is that uncivil behaviours will spill into practice affecting not only working relationships but also patient care. (Clark & Springer, 2010).

## 2 BACKGROUND

Nursing students' experiences of incivility behaviours in the clinical setting have been reported to be different. Natarajan (2017) in Oman found that the prevalence of incivility behaviours was moderate, and more than 40% of nursing students had experienced various forms of incivility behaviours including disrespect, cell phone use, being late and boredom in the classroom (Natarajan et al., 2017). Woodworth, (2016) also stated that the behaviour learned by nursing students in nursing schools is transferred to the work environment and nursing culture (Woodworth, 2016). Clark & Springer (2007) showed that more than 70% of participants believed that incivility behaviour in nursing education is a serious problem.(Clark & Springer, 2007). Kim (2018) in South Korea reported that the level of incivility behaviours experienced by students in the clinical setting was moderate (Kim, 2018). Rieck (2007) in the United States found that 35% of nursing students experienced the incivility behaviours from colleagues, and 60% on the part of clinical educators (Rieck & Crouch, 2007). Several studies have identified nursing staff in the clinical areas as a contributing factor to stress and anxiety among nursing students (Levett‐Jones, Lathlean, Higgins, & McMillan, 2009; Moscaritolo, 2009). Anthony & Yastik (2011) in the United States found that incivility towards nursing students does occur and has an important effect on student experiences in the clinical sitting (Anthony & Yastik, 2011).

Therefore, the incivility behaviours during nursing education can be effective in shaping the professional role of nursing students (Palaz, 2013). However, nursing professionalism affects nursing work and nurses' status and leads to the formation of nursing professional values during nursing training in clinical settings (Bang et al., 2011). Professional values are criteria for practice that are accepted by occupational groups and provide a standard framework for evaluating the values, perspectives and beliefs influencing the behaviours (Fahrenwald et al., 2005). The development of professional values in nursing is very important due to the quality of care and increasing patient understanding. It also contributes to the process of professional socialization of students (Donmez & Ozsoy, 2016). In this regard, Khadjehturian (2012) showed that incivility behaviours in clinical education impair students' ability to become professional (Khadjehturian, 2012). Kim in Korea found a statistically significant negative correlation between the experience of incivility behaviours and nursing professional values (Kim, 2018).

However, limited studies have examined the correlation between incivility behaviours and nursing professional values among nursing students in the world, including Iran. The degree of generalization of the results of previous studies on incivility behaviours and nursing professional values to other countries and nursing students has not yet been well studied. Yet, the difference in the nursing education system and culture of different countries justifies conducting a study among Iranian nursing students in order to give new information and insights in this field to nursing policymakers and managers in Iran. Therefore, this study aimed to investigate the correlation between incivility behaviours and professional values among nursing students.

## 3 DESIGN

### 3.1 Method

#### 3.1.1 Participants and research context

The present study was conducted between August and December 2019 on 400 nursing students who were taking clinical courses (apprenticeship) in teaching hospitals. Inclusion criteria were nursing students who had completed at least one clinical course or apprenticeship in a hospital. Guest students from other universities were excluded from the study. According to a study by Kim in 2018.,(Kim, 2018) the sample size (n) was calculated 384 people using the equation of infinite sample size estimation (n = [z2 * p * q]/d2) and with a 95% confidence interval (*α* = .05), Z score 1.96 and accuracy (d) of 0.05. Further, 10% was considered for dropout or incompleteness of the questionnaire. Therefore, 422 questionnaires were distributed among students by convenience sampling method. Participation was voluntary, and the participants were informed of the research objectives, voluntary participation, anonymous responses, and confidentiality terms about their personal information. In addition, written informed consent was obtained from the selected nurses' students prior to enrollment. Finally, with a response rate of 94.8%, 400 valid questionnaires were included into the analysis. High response rate is probably driven by exact explanation about the research by researcher, before fill in the forms.

#### 3.1.2 Data collection

Data gathering tools used in the study includes demographic characteristics, incivility behaviour, and nursing professional values. Demographic profiles included age, gender, marital status, work experience, nationality, ethnicity, and academic year. The Uncivil Behaviour in Clinical Nursing Education (UBCNE) questionnaire was developed by Anthony & Yastik in 2011 and revised in 2014 (Anthony et al., 2014). The questionnaire consists of 2 dimensions and 12 items. The “hostile/mean/dismissive” dimension includes 7 items and the “exclusionary behaviour” dimension has 5 items, which together indicate the total score of incivility behaviours. The UBCNE items were rated as 5‐point Likert scale (0 = never, 1‐rarely, 2 = sometimes, 3‐often and 4 = always). The score of incivility behaviour is obtained from the score of its total dimensions. The scores of this questionnaire are between 0 and 4. The higher the total score, the higher the incivility behaviours of nurses towards students. Cronbach’s alpha coefficient for total score of incivility behaviours, hostile/mean/dismissive and exclusionary behaviour dimensions were 0.81, 0.88 and 0.83, respectively (Anthony et al., 2014). The Nursing Professional Values Scale (NPVS) was designed by Weis & Schank (2017) to assess nurse’s professional values (Weis & Schank, 2017). The scale includes 26 items in five dimensions of caring (9 items), activism (5 items), trust (5 items), professionalism (4 items) and justice (3 items). Items are answered on a 5‐point Likert scale (1 = “not important” to 5 = “very important”). The total score of the scale is between 1 and 5. Higher scores indicate higher nursing professional values. The validity and reliability of NPVS have already been fully reported by Bouzaripour et al., and the Cronbach’s alpha coefficient for total score of NPVS was 0.94 (Boozaripour, Abbaszadeh, Shahriari, Hashiani, & Borhani, 2017).

#### 3.1.3 Data analysis

Data were analysed using IBM SPSS version 21. Demographic characteristics of the research units, incivility behaviours, nursing professional values and their dimensions were described by descriptive statistics such as frequency, percentage, mean and standard deviation. In the next step, the data were examined for normality using the Kolmogorov–Smirnov test, which showed the normal data distribution. Pearson correlation coefficient (r) was performed to determine the nature of the correlation between the incivility behaviours and the nursing professional values. Multiple linear regression analysis was performed to identify the predictive value of the dependent variable (incivility behaviour) by the independent variable (nursing professional values). Demographic variables were included in the model as possible confounding factors. The tests were performed at a significance level of 0.05.

## 4 RESULTS

### 4.1 Demographic characteristics of the participants

The mean age of participants was 22.35 years with a standard deviation of 3.32 years. The majority of respondents were women (60.7%) and single (86.5%) (Table 1).

**TABLE 1:** Demographic characteristics of nursing students (*n* = 400)

Demographic characteristics	No.	Percentage (%)
Age in years (Mean = 22.35; SD = 3.32)		
18–21	200	50.0
22–30	187	46.7
>30	13	3.3
Gender		
Female	243	60.7
Male	157	39.3
Marital status		
Single	344	86.5
Married	54	13.5
Work experience		
Yes	101	25.3
No	299	74.7
Ethnicity		
Fars	179	44.8
Kurd	55	13.8
Turk	75	18.8
Lor	49	12.3
Others	42	10.5
Nationality		
Iranian	395	98.7
Non‐Iranian	5	1.3
Academic year		
1st year	115	28.75
2nd year	85	21.25
3rd year	109	27.25
4th year	91	22.75

### 4.2 Incivility behaviours and nursing professional values

Table 2 shows the mean and standard deviation of incivility behaviour scores experienced by students from nurses, perceived professional values and their dimensions. Based on the results, the mean score of experienced incivility behaviours was 1.76 out of 4. The highest and lowest mean scores of different dimensions of incivility behaviours experienced were related to lack of cooperation (2.20) and violence or disrespect/ignorance (1.33), respectively. In addition, the mean total score of nursing professional values among students was 3.78 out of 5. Among the dimensions of professional values, the maximum and minimum scores were related to the dimensions of caring (3.84) and activism (3.63), respectively.

**TABLE 2:** Mean and standard deviation (SD) of the obtained scores of incivilities, nursing professional values and the associated dimensions (*n* = 400)

	Mean ± SD	Observed range
Trust	3.65 ± 0.86	1–5
Justice	3.80 ± 0.91	1.33–5
Professionalism	3.69 ± 0.92	1–5
Activism	3.63 ± 0.86	1–5
Caring	3.84 ± 0.87	1–5
**Total NPVS**	3.72 ± 0.80	1.40–5
H‐M	1.33 ± 0.75	0–4
Exclusionary behaviour	2.20 ± 1.04	0–4
**Total incivility behaviour**	1.76 ± 0.80	0–4

H‐M, hostile/mean/dismissive; NPVS, nursing professional values.

### 4.3 Correlation between incivility behaviours and professional values

According to Table 3, Pearson correlation coefficient shows a statistically significant negative correlation between the experienced incivility behaviours and the total score of professional values (*r* = −0.150, *p* = .003). There was also a statistically significant and negative correlation between incivility behaviours and dimensions of “justice,” “activism” and “caring” (*p* < .01), while no statistically significant correlation was found between incivility behaviours and the dimensions of “trust” and “professionalism” (*p* > .05).

**TABLE 3:** Correlation between experienced incivility behaviour and its dimensions and nursing professional values and its dimensions in research participants (*n* = 400)

Variables	H‐M		Exclusionary behaviour		Incivility behaviour	
	*R*	*p*	*r*		*r*	*p*
Trust	−0.233	<0.001**	−0.032	0.527	−0.089	0.077
Justice	−0.284	<0.001**	−0.004	0.941	−0.135	0.007**
Professionalism	−0.219	<0.001**	−0.031	0.531	−0.123	0.014
Activism	−0.256	<0.001**	−0.068	0.177	−0.164	0.001**
Caring	0.255	<0.001**	−0.07	0.164	−0.165	0.001**
Total NPVS	−0.277	<0.001**	−0.031	0.535	−0.150	0.003**

Value of Pearson correlation coefficient: *r* = 0.1 weak relationship; *r* = 0.3 moderate relationship; *r* = 0.5 strong relationship. *p < 0.05; **p < 0.01.

Table 4 shows the results of multiple linear regression to evaluate the predictive value of the nursing professional values via the incivility behaviours by controlling demographic variables. The value of regression coefficient between incivility behaviour as a dependent variable and professional values as an independent variable was *R* ^2^ = .05, which means that incivility behaviours have a statistically significant role in predicting nursing professional values (*F* = 2.92, *p* = .005). Among the demographic variables, gender was significantly correlated with uncivil behaviours, so that female students experienced more incivility behaviours by nurses than male students (B = 0.235, 95% CI 0.077–0.394, *p* = .004).

**TABLE 4:** Multivariate regression analysis between incivility and nursing professional values (*N* = 400)

Variables	B	SE	95% CI	*p*
Age (reference: >30)				
18–21	0.272	0.238	[−0.195 to 0.739]	.254
22–30	0.311	0.232	[−0.143 to 0.766]	.180
Marital status (reference: Married)				
Single	−0.064	0.122	[−0.304 to 0.175]	.599
Gender (reference: Male)				
Female	0.235	0.081	[0.077 to 0.394]	.004
Nationality (reference: Iranian)				
Non‐Iranian	−0.103	0.352	[−0.794 to 0.588]	.770
Ethnicity (reference: Others)				
Fars	−0.116	0.135	[−0.38 to 0.149]	.390
Kurd	−0.056	0.160	[−0.370 to 0.258]	.727
Turk	0.060	0.152	[−0.237 to 0.357]	.692
Lor	−0.027	0.165	[−0.351 to 0.297]	.870
Total NPVS	−0.153	0.049	[−0.249 to −0.056]	.002

*R* = 0.223, *R* ^2^ = 0.05, *F* = 2.92, *p* = .005

## 5 DISCUSSION

The aim of this study was to determine the correlation between incivility behaviours experienced by nursing students from nurses and nursing professional values in hospitals. Findings showed that the incivility behaviours experienced by students from nurses are below average (score 1.7 out of 4). In this regard, Kim (2018) estimates that the mean total score of incivility behaviours experienced are below average (Kim, 2018). Natarajan et al. (2017) in Oman reported that the prevalence of incivility behaviours was moderate (Natarajan et al., 2017), while a qualitative study in Iran showed that all participants experienced incivility behaviours on the part of nurses (Rafati, Nouhi, Sabzevar, & Nayyeri, 2017). Zahedi et al. (2013) in Iran examined the codes of nursing ethics, and showed that one of the ethical codes of nurses in Iran that should be given more attention is that “nurses should have a respectful behaviour and attitude towards other nurses, teachers and students” (Zahedi et al., 2013).

Concerning the nursing professional values, it was observed that the level of nursing professional values is above average (3.72 out of 5). In line with the findings of the present study, Boozaripour et al. (2017) in Iran used the NPVS tool among nursing students, and reported that the mean perceived nursing professional values is higher than average (Boozaripour et al., 2017). The mean score of professional values perceived by nursing students in a study conducted by Donmez & Ozsoy (2016) in Turkey was reported to be 3.82, which is higher than average in general (Donmez & Ozsoy, 2016). Kobra et al. (2012) reported that the mean perceived nursing professional values were above average (Kobra, Vahid, & Alsadat, 2012).

In this study, the activism dimension received the lowest score and these findings were in line with the results of previous studies (Boozaripour et al., 2017; Donmez & Ozsoy, 2016), while another study of US nursing students by Alfred et al. (2013) found that the most important dimension is activism (Alfred et al., 2013).

The caring dimension in this study had the highest mean among the respondents. Watson & Smith believe that patient care is a fundamental component of holistic nursing care (Watson & Smith, 2002). However, Bang et al. (2011) showed that the most important dimension for South Korean nursing students was professionalism (Bang et al., 2011). The professionalism in nursing is related to the cultural and educational structures of a society. Therefore, many efforts are needed to improve the image of nursing, and nursing educators need to address these issues in clinical settings in dealing with nursing students so that they can empower their students to meet these challenges (Bang et al., 2011).

The results of this study revealed a negative and statistically significant correlation between incivility behaviours experienced by students and nursing professional values. The findings also showed that nursing professional values could be experienced as a predictor of incivility behaviours. The regression coefficient showed that 5% of the explained variance of nursing professional values is negatively estimated by incivility behaviours. Although correlation and regression values were low in the present study, it seems that adherence to nursing professional values can affect the incivility behaviours. Given this negative and statistically significant correlation, it can be concluded that the reduction in the incivility behaviours from nurses as patterns of the nursing profession causes nursing students to better acquire and internalize nursing professional values in the clinical setting. Kim (2018) showed that the experienced incivility behaviours were negatively correlated with nursing professional values (Kim, 2018). Previous studies have shown that nursing students derive professional values directly and indirectly from positive role patterns in their clinical setting (Lúanaigh, 2015; Shafakhah, Molazem, Khademi, & Sharif, 2018). Thomas et al. (2015) stated that nursing students in clinical settings can develop their professional roles and nursing professional values (Thomas, Jinks, & Jack, 2015). Ham (2004) pointed out that the growing ethical conflicts in the field of caring have raised the need to pay attention to professional values (Ham, 2004). In the clinical setting, the feeling of support and respect from nurses can establish a positive image of the nursing profession among nursing students (Wallace et al., 2015).

## 6 STUDY LIMITATIONS

Although this study was the first to be conducted in Iran, it had several limitations. First, our research samples were selected from the same nursing school in Tehran. Therefore, it is suggested that researchers should conduct a study in other colleges with larger sample size to promote the generalization of the study results. In addition, since the nursing professional values are influenced by many individual and environmental factors, the use of a structured self‐report questionnaire may limit our understanding of these values. Therefore, it is better to design a qualitative study for a deep understanding of nursing professional values.

## 7 CONCLUSION

This study aimed to investigate the correlation between experienced incivility behaviours by nursing students and professional values. The findings of this study showed that the level of incivility behaviours experienced by students was low and the nursing professional values were moderate to high. The findings of this study also confirmed that nurses' incivility behaviours towards students have a negative effect on nursing professional values.

## AUTHOR CONTRIBUTIONS

VN, MB, FA: study concept; VN and PE: data collection; MB and FA: data analysis and interpretation; VN, MB, FA and PE: manuscript drafting; VN and MB: statistical analysis;: MB, FA and PE: study supervision. All authors contributed to the writing of the manuscript.

## FUNDING INFORMATION

This research received no specific grant from any funding agency in the public, commercial or not‐for‐profit sectors.

## CONFLICT OF INTERESTS

No conflict of interest has been declared by the authors.

## ETHICAL STATEMENT

This research was approved by the ethics committee of Faculty of Nursing and Midwifery, Shahid Beheshti University of Medical Sciences, Tehran. Ethics Approval Number: IR.SBMU.PHARMACY.REC.1398.132.

## Data Availability

The data that support the finding of this study are available on request from the corresponding author. The data are not publicly available due to privacy or ethical restrictions.
